# Developmental profile of tissue plasminogen activator in postnatal Long Evans rat visual cortex

**Published:** 2008-05-28

**Authors:** Sha Zheng, Zheng Qin Yin, Yu Xiao Zeng

**Affiliations:** Southwest Eye Hospital, Southwest Hospital, Third Military Medical University, Chongqing, China

## Abstract

**Purpose:**

To investigate the distribution, expression, and activity of tissue plasminogen activator (tPA) in the visual cortex of the Long Evans rat during postnatal development, and to explore the relationship between tPA levels and the critical period of visual cortical plasticity.

**Methods:**

Long Evans rats of either sex (n=131) were divided by postnatal age in weeks (PW) into five groups: PW1 (6–8 days, before eye opening, n=19), PW3 (20–22 days, beginning of critical period, n=28), PW5 (34–36 days, later stage of critical period, n=28), PW7 (48–50 days, end of critical period, n=28), and PW14 (95–100 days, adult, n=28). The distribution and expression of tPA was detected using immunofluorescence histochemistry and western blot analysis, respectively. tPA activity in the visual cortex was determined using a chromogenic assay kit.

**Results:**

tPA-containing cells were mostly located in visual cortex layer II-III and layer IV during postnatal development. In layer II-III the density of tPA-containing cells reached peak at PW 5, and then reduced to minimum at PW14. In layer IV and V-VI, the density of tPA-containing cells reached a maximum at PW3, and then decreased to the minimum at PW14. Western blot analysis indicated that tPA was detected in visual cortex of rats from PW3 onwards with the highest quantity present at PW5. By comparison, the peak in tPA activity occurred slightly earlier at PW3, and then decreased steadily to lower levels at PW14.

**Conclusions:**

The critical period of visual cortical plasticity, which occurs in early postnatal life, correlates well with tPA expression in the rat visual cortex. This suggests that the expression of tPA is produced in sufficient amounts to balance the increase of chondroitin sulfate proteoglycan expression, at the same time blocking its function, thus allowing synaptic modification to continue. tPA activity may be one of the factors influencing the duration of the critical period and underlying the heterogeneity of synaptic plasticity between visual cortex layer II-III and layer IV.

## Introduction

The intrinsic neural connections and synaptic structure of the visual system can be regulated and modified postnatally by stimulation from the visual environment. This phenomenon occurs in a well defined period called the critical period of visual cortical plasticity [1-3]. The critical period extends from postnatal 3 months to 6 years of age in humans, and from postnatal 3 to 6 weeks in rats [4]. Once this critical period has passed, the presence of an abnormal visual environment cannot lead to amblyopia, and existing amblyopia is difficult to rectify. There has not been any effective drug treatment method devised to date that can ameliorate the effects of amblyopia. Therefore, investigation of the mechanisms leading to the termination of cortical plasticity in developing mammals will help establish a new approach for reestablishing visual function in older amblyopic children and adults. Elucidation of the possible opening or closing-factor(s) may also supply an experimental basis for drug treatment of amblyopia.

Tissue plasminogen activator (tPA) is a key molecule in the regulation of the extracellular proteolytic cascade reaction in the postnatal mammalian central nervous system [5]. Recent studies suggest that during the critical period of visual cortical plasticity, tPA may influence synaptic reconstruction by its proteolytic effect on chondroitin sulfate proteoglycans (CSPGs), which are major components of the extracellular matrix (ECM) [6]. However, it is not known whether tPA activity is correlated with the initiation or termination of the critical period.

To explore the relationship between the temporal/spatial profile of tPA and the duration of the critical period of visual cortical plasticity, we used immunofluorescence histochemistry, western blot, and chromogenic assays to investigate the distribution, expression, and activity of tPA in the visual cortex of postnatal Long Evans rats.

## Methods

All Long Evans rats used in this study were treated according to Chinese law on the use of laboratory animals, and followed the ethical guidelines of the Laboratory Animal Care and Use Committee of ARVO.

Long Evans rats (n=131; Taconic Farms, Hudson, NY) of either sex were divided into five groups according to postnatal age in weeks (PW): PW1 (6–8 days, before eye opening, n=19), PW3 (20–22 days, beginning of critical period, n=28), PW5 (34–36 days, later stage of critical period, n=28), PW7 (48–50 days, end of critical period, n=28), and PW14 (95–100 days, adult, n=28). All rats were housed in a specific pathogen free (SPF) animal room under normal light-dark conditions (12-h light/dark cycle), and given regular rat chow and clean water ad lib.

### Immunohistochemistry and analysis of cell density

Animals (PW1, PW3, PW5, PW7, and PW14, n=9 for each group) for immunofluorescence histochemical analysis were euthanized with 200 mg/kg ketamine i.p. (HengRui Medical Ltd, Jiang Shu, China) and then transcardially perfused with saline followed by 4% paraformaldehyde in 0.1 M phosphate buffer (pH 7.4). Brains were removed, post-fixed overnight at 4 °C, and then tissue blocks were equilibrated in 30% sucrose for cryoprotection. A series of frozen coronal sections of the visual cortex (extending ~1–3 mm from the occipital poles) were cut at 30 µm with a freezing microtome and collected in 0.01 M phosphate-buffered saline (PBS; pH 7.4). Free-floating sections were rinsed at least three times for 10 min with PBS, then potential nonspecific binding sites were blocked for 1 h at 37 °C with 5% normal goat serum in PBS containing 0.3% Triton X-100 (Sigma-Aldrich, Shanghai, China). The sections were incubated with rabbit antimurine tPA antibody (catalog number 1188; American Diagnostica Inc., Stamford, CT) overnight at 4 °C. Exclusion of the primary antibody was used as an internal control. After rinsing in PBS, the sections were processed with a secondary antibody, goat anti-rabbit IgG-FITC (sc-2012; Santa Cruz Biotechnology, Santa Cruz, CA) for 1 h at 37 °C. Finally, the tissue was extensively washed with PBS, briefly placed in distilled water, mounted on fluorescence-free slides, air-dried, and coverslipped with Entellan.

**Figure 1:**
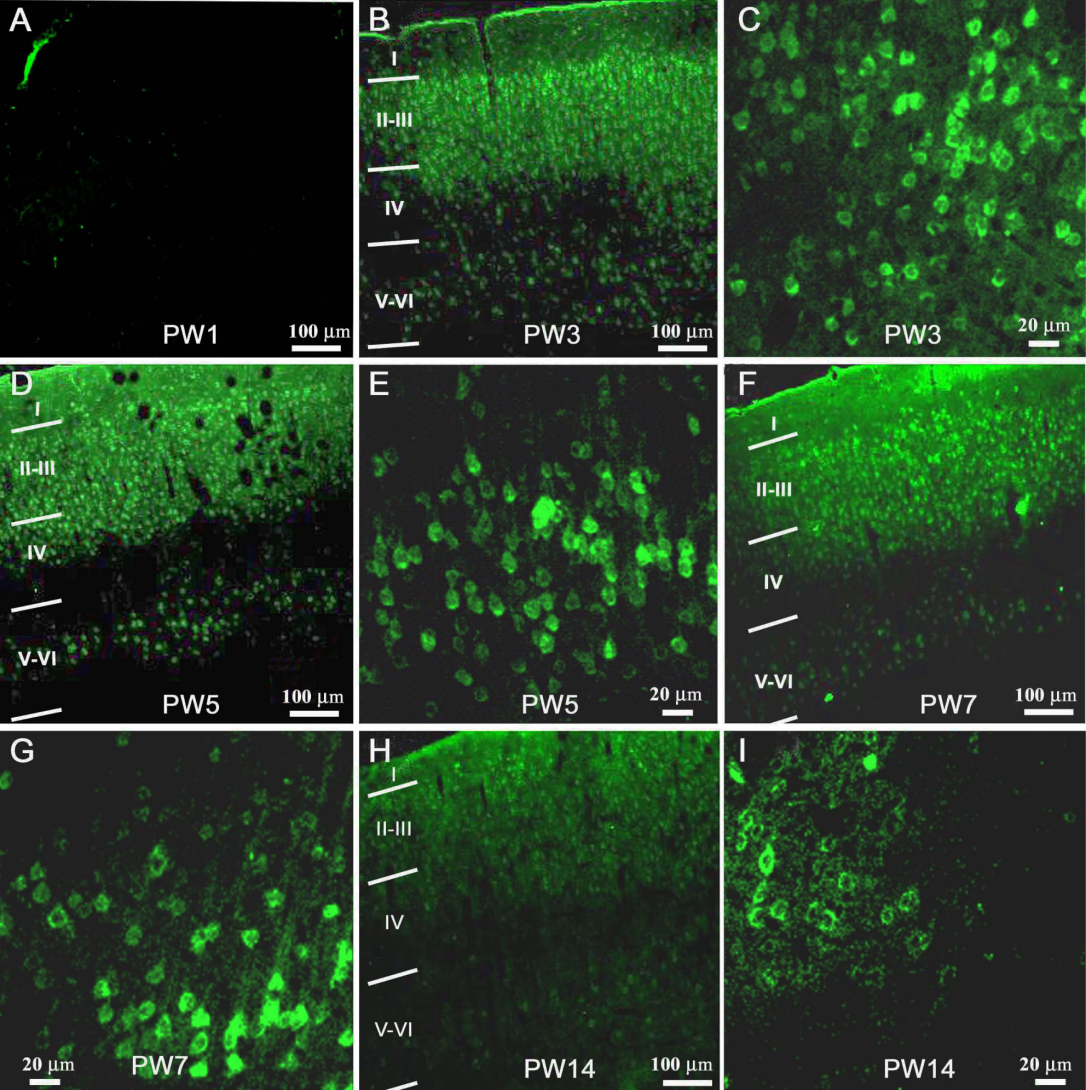
Tissue plasminogen activator immunoreactivity in the visual cortex of the Long Evans rat. **A**, **B**, **D**, **F**, and **H**: Low magnification photomicrographs shows the visual cortices of rats aged postnatal week 1 (PW1), PW3, PW5, PW7, and PW14, respectively. Photomicrographs were taken in the transitional region between monocular and binocular visual cortex. Note that no tissue plasminogen activator (tPA) is stained in the visual cortex at PW1 (**A**), although tPA-positive cells are prominent in layer II-III at later ages. **C**, **E**, **G**, and **I**: Higher magnification photomicrographs shows tPA staining in layer V-VI at the same ages. Note the faint apical dendritic labeling seen at PW3, PW5, and PW7. The scale bar in **A** represents 100 μm and also refers to **B**, **D**, **F**, and **H**; the scale bar in **C** represents 20 μm and also refers to **E**, **G** and **I**.

The slides were inspected using a confocal laser scanning microscope (Leica TCS 4D; Leica Microsystems Wetzlar GmbH, Wetzlar, Germany or Zeiss LSM 510; Advanced Imaging Microscopy, Jena, Germany) with excitation parameters for fluorescein isothiocyanate (FITC; 528 nm). Fields from sections of visual cortex stained with tPA were acquired from layer II-III (depth <350 µm), layer IV (depth between 350 and 500 µm), and deep layer V-VI (depth >500 µm). The full thickness of the visual cortex was roughly similar at all ages examined, and, although the thickness of layer I was thinner at later ages, the depth of the other layers changes very little. In this regard, we analyzed cell densities at cortical depths that correspond to those used by Pizzorusso and coworkers [7], thus making the studies comparable.

The analysis was performed on three sections from each of the nine animals at the different ages (PW3, PW5, PW7, and PW14). Image Proplus 4.5 was used to quantify the density of tPA-labeled cells by counting labeled cells within three sample boxes (each box=625 µm^2^) in each of three regions (i.e., layers II-III, VI, V-VI). The shape of the sample box was adjusted while maintaining the sample area to include the full dorsoventral thickness of the layer counted. To determine the number of labeled cells across the full thickness (all layers) of the cortex, we counted all labeled cells within a 250 µm strip extending from the cortical surface to the bottom of layer V-VI.

### Western blot analysis of tissue plasminogen activator

The primary visual cortices (lateral 2–4 mm, posterior 0–2 mm) from animals (PW1, PW3, PW5, PW7, and PW14, n=10 for each group) were dissected away from the underlying white matter with a microdissecting knife and the aid of a Zeiss Stemi DV4 dissecting microscope and transillumination. The tissue from two animals for each sample at each age was then homogenized in RIPA buffer (1% Triton/0.5% sodium deoxycholate/0.1% SDS/PBS/2 mM DTT) and after sonication and centrifugation, the supernatants were collected and protein concentrations determined using a modified Lowry method [8]. Matching amounts of protein from each sample were mixed with sample buffer. Samples were loaded onto a 7.5% SDS–PAGE linear gel. Following electrophoresis, gels were blotted to a polyvinylidene fluoride (PVDF) membrane. Membranes were blocked with 5% nonfat milk for 2 h, then incubated overnight at 4 °C with 1:500 primary antibodies to tPA and 1:10000 glyceraldehyde-3-phosphate dehydrogenase (GAPDH; catalog number KC-5G4; Kang Chen, Shanghai, China). Subsequently, PVDF membranes were blocked again and incubated with antirabbit (1:2000) and antimouse (1:2000) alkaline phosphatase (AP) conjugated secondary antibodies (Sigma, Saint Louis, MO) for 1 h at room temperature. The bands were visualized by the chromogenic substrate 5-bromo-4-chloro-3′-indoly-phosphate/nitro-blue tetrazolium chloride (BCIP/NBT; Pierce, Rockford, IL) and the mean density quantified using the “measure” function of the Quantity One program, Version 4.4. GAPDH was used as a loading control for western blot and protein normalization [9]. We optimized the western blotting experimental protocols to avoid background band staining and make the target binding of the single band of tPA labeling at 67 kDa and that of GAPDH distinct. Our improved protocols were based on methods of Zhao and colleagues [10].

### Proteolytic chromogenic assay of tissue plasminogen activator

Animals (PW3, PW5, PW7, and PW14, n=9 for each group) for proteolytic chromogenic assay of tPA were euthanized by inhalation of 5% volatile halothane, and then quickly perfused transcardially with ice-cold PBS. The visual cortices were removed and weighed. After homogenization with a buffer: consisting of 20 mM PBS, 320 mM sucrose, 1 mM EDTA, and 0.2% Tween20, followed by centrifugation at 4 °C, supernatants were collected and tPA activity was determined by a chromogenic assay kit (Department of Molecular Genetics, Shanghai Medical University, Shanghai, China). Activity was measured by adding Glu-plasminogen, chromogenic plasmin substrate, and fibrin at pH 7.4. In the presence of fibrin, tPA converts plasminogen to plasmin, which subsequently cleaves the chromogen substrate. The color absorbance (yellow) developed over 4 h at 37 °C and measured at 405 nm was proportional to the amount of tPA activity in the cortical homogenate.

All population data are expressed as the mean *±* standard deviation. The data were evaluated by one-way ANOVA followed by a least significant difference (LSD) multiple comparison *t*-test.

## Results

### Immunohistochemistry and analysis of cell density

**Figure 2:**
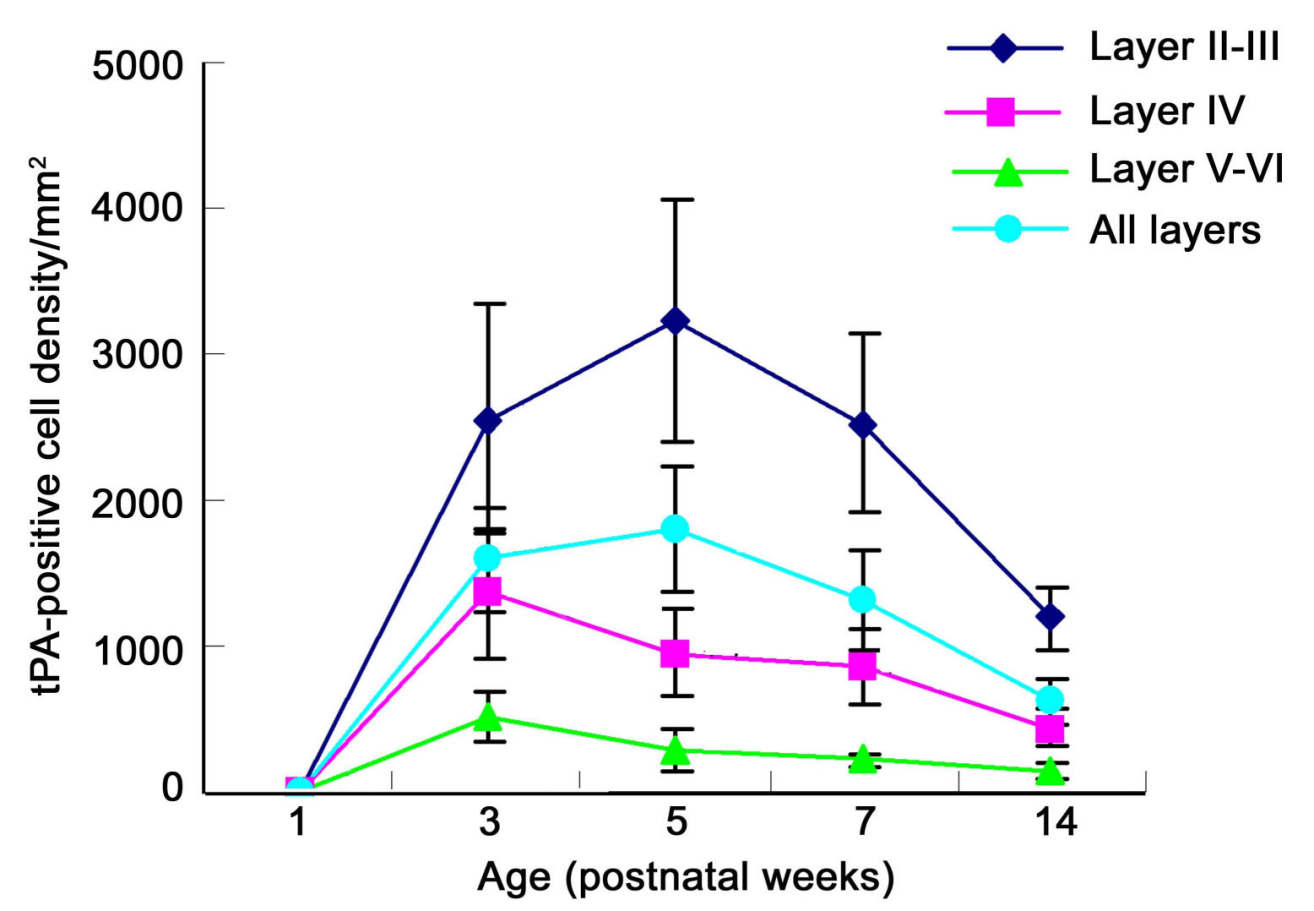
Distribution and profile of tissue plasminogen activator (tPA)-positive cell density in visual cortex during post-natal development. tPA was visualized with a fluorescein isothiocyanate (FITC)-conjugated secondary antibody and the number of cells within each layer counted (data points represent the mean±SD, n=9). Data was compared using a least significant difference-(LSD) multiple comparison *t*-test. tPA labeling is prominent in layer II-III and layer IV. Comparisons between layers showed that labeled cell density in layer II-III was significantly higher than layer IV and V-VI (p<0.01) and cells density in layer IV was higher than that in layer V-VI (p<0.05). Within layer II-III, tPA labeled cell density reaches a maximum at PW5, and then declines, such that the following cell density comparisons were significant; PW3 versus PW5 and PW14 (p<0.05, 0.01, respectively), PW5 versus PW7 and PW14 (p<0.05, 0.01, respectively), and PW7 versus PW14 (p<0.01), respectively. Note that in layer IV the density of tPA labeled cells reaches a maximum at PW3 that is significantly higher than values at PW5, PW7, and PW14 (p<0.01), similarly values at PW5 and PW7 are higher than at PW14 (p<0.01). The same trend was seen within layer V-VI and labeled cell density at PW3 significantly higher than at later ages (p<0.01). The density of tPA-containing cells across the whole thickness of the visual cortex reaches a maximum at PW5 and declines significantly to lower densities at PW7 before reaching a minimum at post-natal PW14 (PW14 versus PW3, PW5, and PW7, p<0.01). Cell density at PW5 was significantly higher than that at PW7 (p<0.05).

tPA immunolabeling was located in the cellular membrane and cytoplasm of positively stained cells of the visual cortex (Figure 1). No tPA staining was present in the visual cortex of PW1 animals (Figure 1A), but was clearly present by PW3 (Figure 1A,B) and prominent in layer II-III; the highest cell density of tPA labeled cells were located in layer II-III (p<0.01; see Figure 1B,D,F,H and Figure 2). Figure 2 compares the changes of labeled cell density between cortical layers at the different ages. Note that the peak changes occur during the critical period of cortical plasticity in the rat, i.e., between two and six weeks. In layer II-III, the density of tPA-containing cells reached a maximum at PW5 (3231.7±836.2/mm^2^), which was significantly higher compared to all other ages (PW5 versus PW3 and PW7, p<0.05; PW5 versus PW14, p<0.01), and then was reduced to a minimum by PW14 (PW14 versus PW3 and PW7, p<0.01; see Figure 2, layer II-III). In layer IV and layer V-VI (Figure 2), the density of tPA-positive cells reached a maximum at PW3 (1358.9±451.2/mm^2^ and 512.0±179.2/mm^2^, respectively). For both layer IV and layer V-VI, densities of labeled cells at PW3 were significantly higher than at later ages (layer IV, PW3 versus PW5–14, p<0.01; layer V-VI, PW3 versus PW5–14, p<0.01). In layer V-VI, the density of labeled cells declined at PW5 (280.4±138.8/mm^2^), reaching a minimum at PW14 (139.1±65.9/mm^2^). There were no significant differences between PW5-PW14. As shown in Figure 2, in layer IV, the density of cells at PW14 (438.0±130.1/mm^2^) was significantly lower (p<0.01) than that seen at PW5 (952.8±297.2/mm^2^) and PW7 (858.0±264.0/mm^2^). The average density of tPA-containing cells across the whole thickness of visual cortex reached a peak density by PW5 (1792.5±423.4 /mm^2^), which was significantly reduced by PW7 (1302.4±342.6/mm^2^; PW5 versus PW7, p<0.05) with a further decline in numbers to a minimum at PW14 (619.2 ± 158.9/mm^2^; PW14 versus PW3–7, p<0.01).

### Western blot analysis of tissue plasminogen activator

**Figure 3:**
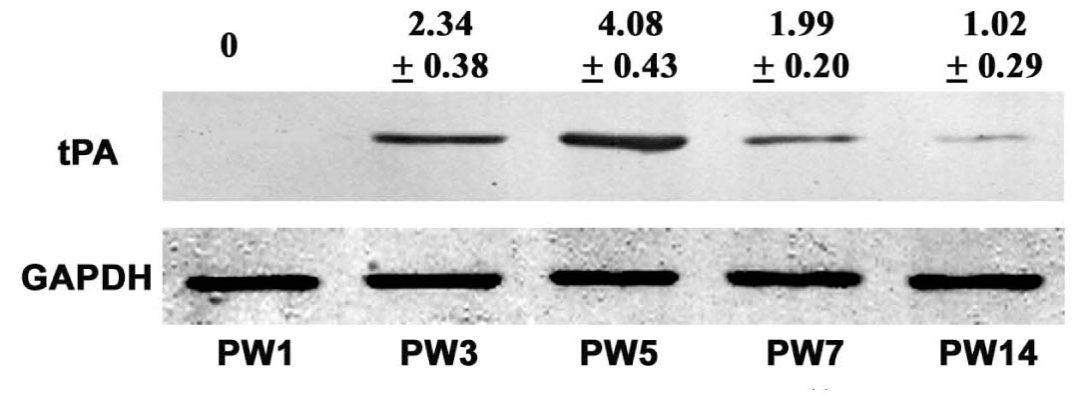
Western blotting for tissue plasminogen activator (tPA) expression in the visual cortex during post-natal development. tPA has a molecular weight corresponding to 67 kDa. The staining protocol was optimized to produce a single distinct band. Data at the top of the bands shows the tPA/glyceraldehyde-3-phosphate dehydrogenase (GAPDH) ratio (%) at each age (mean±SD, n=10). Microdensitometry revealed that tPA is not expressed at PW1 but is present at all other ages sampled. Comparisons were made using a least significant difference (LSD) multiple comparison *t*-test. tPA reaches its highest expression at PW5 and then declines. Protein expression at PW3, PW7, and PW5 was significantly higher than at PW14 (p<0.05 and 0.01 respectively). The peak value at PW5 was significantly higher than that seen at PW3 and PW7 (p<0.05).

tPA was detected in the visual cortex of all but the PW1 rats; animals at PW5 (4.08%±0.43%, p<0.05) showed the highest values (expressed as a function of optical density measurements; see Figure 3). This peak in the quantity of tPA seemed to occur at a time when tPA cell density in layer II-III reached its maximum (Figure 2 and Figure 3). Similar to the data presented for cell density in the different layers of the cortex, animals at PW14 had the lowest tPA expression (1.02%±0.29%, p<0.05; Figure 2 and Figure 3). Note that activity is clearly highest over the period of PW 3–5, coinciding with the cortical critical period.

### Proteolytic chromogenic assay of tissue plasminogen activator

**Figure 4:**
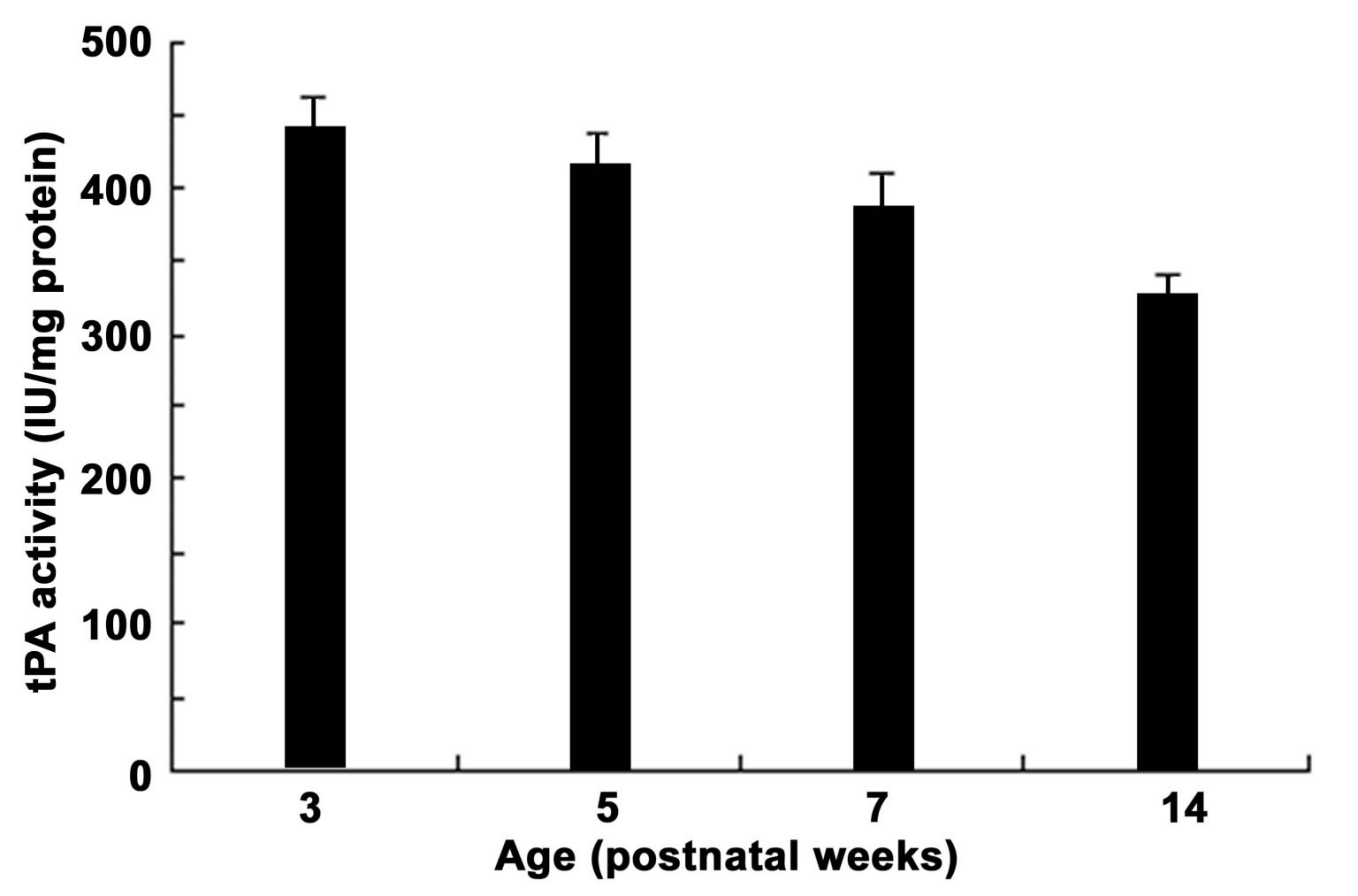
Tissue plasminogen activator (tPA) activity of the visual cortex during postnatal development. Histograms show tPA activity measured as a function of the conversion of plasminogen to plasmin by tPA. Each bar represents the mean±SD (n=9). tPA activity is highest at PW3, and then declines at later developmental stages with the lowest value at PW14. Values at PW14 were significantly lower compared to all other ages (p<0.01). Activity at PW3 was not significantly different from that at PW5 but was higher than that at PW7 (p<0.01). Values were compared using a least significant difference (LSD) multiple comparison *t*-test.

tPA activity peaked at PW3, and this peak occurred somewhat earlier when compared to the peak in tPA expression shown on the western blot at PW5 (Figure 3 and Figure 4). This peak in the conversion of plasminogen to plasmin coincided with the peak cell numbers labeled in layers IV and V-VI (Figure 2 and Figure 4). Similar to the description for cell density and the western blot analysis, the tPA activity was reduced to a minimum by PW14 (323.7±17.1 IU/mg protein; PW14 versus PW3–PW7, p<0.01; Figure 4).

## Discussion

Our experiments show that tPA expression in the rat’s visual cortex is highest over the period of PW3–PW5 and thus coincides with the cortical critical period. The increase in tPA immunoreactivity is particularly prominent in layer II-III by PW5, after which the expression declines at older postnatal ages. The expression and activity of tPA over the critical period was additionally confirmed using western blot analysis and a proteolytic chromogenic assay.

Recently, the role of tPA has come under scrutiny because it is the main serine protease in the central nervous system of postnatal mammals and may play a key role in mediating ocular dominance shifts caused by monocular deprivation [11], a classic paradigm of plasticity. tPA activates its plasminogen substrate, initiating a tPA/plasmin cascade reaction that directly or indirectly degrades a series of ECM molecules. In tPA-knockout mice, ocular dominance shifts caused by monocular deprivation were inhibited, but cortical plasticity could be restored through an upregulation of endogenous or exogenous tPA expression [5]. Mataga et al. [11] suggested that the extracellular microenvironment of the visual cortex may be altered during the critical period due to a local degradation of ECM molecules by the tPA cascade reaction, and thus making it amenable to synaptic structure renewal and reconnection.

### Immunohistochemistry and analysis of cell density

In the present study, the presence of tPA assessed by either immunofluorescence or western blotting was not seen in PW1 rat visual cortex—that is, at a time when the rat pups still have not opened their eyes. tPA-positive staining was detected by PW3 after the rat pups have opened their eyes between postnatal days 12 and 13, indicating that, to some extent, there may be an experience-dependency of tPA expression during the critical period. Current research has made it clear that the synaptic plasticity of layer II-III in visual cortex is greater than that of layer IV, i.e., synaptic plasticity of corticocortical pathways is greater than that of thalamocortical pathways [12]. We found that tPA staining was prominent in layer II-III and layer IV after PW3, with the bulk of the cells being found in layer II-III. This suggests that different tPA distributions between layer II-III and layer IV may reflect differences in the synaptic plasticity between these two layers. Oray et al. [6] examined whether monocular deprivation would occlude a subsequent effect of exogenous tPA/plasmin to determine whether tPA/plasmin might be involved in the structural plasticity of dendritic spines during the critical period. They found that layer II-III, in which spine motility is upregulated by monocular deprivation, was unaffected by additional plasmin application, suggesting that monocular deprivation occluded a further increase in spine dynamics via proteolysis of the extracellular matrix. In contrast, spine motility was significantly increased by enzymatic treatment with plasmin in layer IV, a layer in which spine motility is normally unchanged following deprivation. These results are consistent with the hypothesis that if a selective local secretion of tPA is responsible for the laminar upregulation of spine motility, spines in the middle parts of the apical arbor corresponding to layer IV, will be exposed to less endogenous tPA compared to spines in layer II-III that will be exposed to a much larger amount of endogenous tPA. Our results are consistent with Oray and colleagues [6] and add further weight to this hypothesis. The present data show that during development, the density of tPA-containing cells in layer II-III reaches a maximum toward the latter part of the critical period and is then reduced to minimum after the critical period. By comparison, the density of tPA-containing cells in layer IV reaches a maximum at the peak of critical period, before it is then reduced to minimum at later ages. This prompts the idea that synaptic plasticity of thalamocortical connections may end earlier than that seen for corticocortical connections and this suggestion appears to be supported by the work of Catalano and coworkers [13]. These authors reported that N-methyl-D-aspartate receptor 1 (NMDAR1) expression (necessary for long-term potential [LTP]) in layer IV of the cat visual cortex was rapidly reduced toward the end of the critical period, but its expression in layer II-III remained abundant into adulthood [14].

### Western blot and activity analysis of tissue plasminogen activator

Western blotting for tPA expression and its proteolytic activity illustrates the following points. First, once the critical period ends, tPA expression and activity dramatically decrease, suggesting that CSPGs degradation by tPA is downregulated with a concomitant reinforcement of the inhibition of axon growth and synaptic connectivity by the extracellular microenvironment, thus leading to a reduction in cortical plasticity. Second, lower levels of tPA activity are detected in adulthood, suggesting that tPA may also be involved to some extent in the cortical plasticity seen in adult mammals [15]. Third, the time course of the amount of tPA expression and its corresponding activity were not in complete agreement. Current studies on tPA development in postnatal cerebellum and hippocampus have suggested a region-specific heterogeneity of tPA expression, which may be related to endogenous inhibitors of tPA. In the brain, at least two types of endogenous tPA inhibitors are defined: neuroserpin and nexin-1 [16]. Wannier-Morino et al. [17] reported that during the critical period neuroserpin mRNA was downregulated, while tPA activity was upregulated in the hemisphere contralateral to the deprived eye. Previous investigations concluded that tPA possessing low proteolytic activity is stored in presynaptic vesicles that can be released in a Ca^2+^-dependent pattern after synaptic stimulation [18]. However, concomitant release of endogenous tPA inhibitors would rapidly diminish tPA activity [19]. It has also been suggested that changes in intracellular Ca^2+^ affect dendritic spine morphology, and large increases will cause spine shrinkage and lead to their eventual disappearance [20].

### Possible link to the cortical GABAergic system

Pizzorusso et al. [7] used a chondroitinase ABC enzyme extracted from plants to degrade chondroitin sulfate proteoglycans (CSPGs) in the adult rat visual cortex. They recorded a physiologic shift in ocular dominance after monocular deprivation, indicating that the application had successfully recovered visual cortical plasticity.

This important finding suggests CSPGs may be involved in the termination mechanism(s) for the critical period of cortical plasticity, but also indicates there may be endogenous factors regulating the degradation of CSPGs in the visual cortex that may be relevant to the opening/closure of the critical period. tPA may be one of the key factors for adjusting CSPGs degradation. In contrast to the CSPGs distribution observed by of Pizzorusso et al. [7], in which layer IV has the highest expression and layer II-III and V-VI are roughly similar, in our study, tPA expression is highest in layer II-III and lowest in layer V-VI. The morphology of tPA-labeled cells (note the large lightly labeled dendrites in Figure 1C,E,G; see also [10]) suggests that many of these cells are pyramidal and may account in part for the higher density of tPA-positive cells during the critical period compared to CSPGs cell positive numbers (~10 times more tPA vs CSPGs cells). However, CSPGs and tPA cell number in layer IV-VI in adulthood are very similar [7].

In the visual cortex, the majority of GABAergic inhibitory interneurons are located in layer IV. The GABAergic inhibitory synaptic transmission of visual cortical neurons is strengthened during the critical period, but the trend can be inhibited by binocular form deprivation and recovered by visual input [21]. During the latter stage of the critical period, CSPGs gradually assemble into perineuronal nets (PNNs) around somata and dendrites of GABAergic interneurons [22]. In the visual cortex of adult cats made strabismic as kittens, the density of PNNs, especially in layer IV, is significantly reduced compared to normal animals [23]. Degradation of PNNs reduces the inhibitory function of GABAergic interneurons in layer IV to their target neurons [5], and allows a renewed period of visual plasticity in the adult rat [7]. However, the results of Pizzorusso et al. [7] indicate that the effect of dark rearing is much more pronounced on the distribution of CSPGs-positive cells in layer V-VI compared to layer II-III and layer IV is unaffected (i.e., the layer receiving thalamocortical input). Other findings suggest that the developmental maturation of GABAergic inhibitory neural circuits may be involved in the termination of the critical period [24]. Knockout mice lacking a synaptic isoform of glutamic acid decarboxylase (GAD65) have a prolonged cortical plasticity period, which can be closed by benzodiazepine infusion thus restoring an appropriate level of GABAergic transmission [25]. Similarly, transgenic mice in which the overexpression of brain-derived neurotrophic factor (BDNF) is accelerated show an early closing of plasticity via BDNF’s indirect action on accelerating GABAergic cell development [26]. RNA in situ hybridization showed that overexpression of BDNF associated with the early critical period closure was highest in the superficial layers (layers II and III) [26], that is, in the same layers that show a high convergence and increase in GABA input [27]. Our study found that the density of tPA-positive cells in layer IV-VI progressively decreased during development, with a concomitant downregulation of tPA expression and activity at the closure of the critical period. This suggests that the PNNs containing abundant CSPGs may be the “gate” that can adjust the combination of inhibitory receptors and their isoforms in the cellular membrane with the amount of neurotransmitter. The formation of PNNs may limit synaptic reconnection, resulting in a synaptic structure which is not easily modified; nevertheless, tPA might be one of the keys that can maintain or reopen the “gate.” This hypothesis is supported by our recent in vitro studies on GABA_A_ synaptic transmission in the developing rat visual cortex [28]. tPA-stop significantly increases the peak value and decay time of inhibitory postsynaptic currents compared to normal values seen throughout the critical period. In contrast, addition of tPA to the perfusion solution significantly decreased inhibitory postsynaptic currents peak values and decay times in the cortical slice preparation. These results suggest that tPA or tPA-stop can significantly alter the time course of cortical maturation and the duration of the critical period by altering PNNs and GABA_A_ function.

Both tPA and CSPGs expression increase over the first seven postnatal weeks after which the expression of CSPGs is maintained and that of tPA decreases. This could suggest that the expression of tPA is produced in sufficient amounts to balance the increase of CSPGs expression, at the same time blocking its function, allowing synaptic modification to continue. At the end of the critical period, the shifting balance in favor of CSPGs allows for synaptic consolidation and limits further changes. The degree to which visual activity can control tPA expression remains to be determined. In light of previous CSPGs experiments and dark rearing, we would hypothesize that tPA expression will remain elevated at the end of the critical period as it continues to maintain a balance with CSPGs activity.

Previous reports have shown that several interactions and molecular signals potentially control the length of the critical period [7,11,16,21,29-37]. We have shown that the reduction in tPA is closely correlated with the timing of the critical period of cortical plasticity, and may be one of the factors underlying the heterogeneity in synaptic plasticity between layer II-III and layer IV of the visual cortex. Further research on the specific mechanisms by which tPA contributes to the duration of the critical period and the genetic mechanism(s) that control tPA expression are needed to improve our knowledge of amblyopia pathogenesis and to supply new directions for studying drug treatments for this condition.
